# The Influence of Water/Cement Ratio and Air Entrainment on the Electric Resistivity of Ionically Conductive Mortar

**DOI:** 10.3390/ma12071125

**Published:** 2019-04-05

**Authors:** Ruohong Zhao, Yubin Weng, Christopher Y. Tuan, An Xu

**Affiliations:** 1 International Cooperation in Science and Technology Demonstration Bases for Structural Wind Resistance and Structural Safety, Guangzhou University, Guangzhou 510006, China; rhzhao@gzhu.edu.cn (R.Z.); 2111616188@e.gzhu.edu.cn (Y.W.); xuan@gzhu.edu.cn (A.X.); 2 Department of Civil Engineering, University of Nebraska-Lincoln, Lincoln, NE 68182-0178, USA

**Keywords:** cementitious materials, mortar, air entrainment, electric conductivity, radiant heating

## Abstract

Ionically-conductive mortar can be used for indoor radiant heating partition walls. In these applications, mortar blocks are soaked in electrolyte solutions of CuSO_4_. The surfaces of the block are coated with sealant and epoxy resin afterwards to prevent evaporation. The mortar block becomes a heating element due to ionic conduction if a voltage is applied to the electrodes in the block. Its electrical conductivity depends on the dispersion of the electrolyte, and hence on the porosity of the mortar. The test specimens in this study were divided into four groups according to the different air entrainment agents, including aluminum powder and hydrogen peroxide as well as two air-entraining agents, SJ-2 and K12. Each group was manufactured with water/cement ratios in the range of 0.5 to 0.9. The test results showed that the conductivity of the mortar was strongly influenced by the air-entrainment and the water cement ratios. The volumetric electric resistivity and the associated microstructures of the mortar were investigated. The test results showed that the specimens made with aluminum powder and a water–cement ratio of 0.65–0.75 had high porosity. The porosity of those specimens was further increased by adding two different air-entraining agents. The specimens with aluminum powder and SJ-2, along with a water–cement ratio of 0.7 appeared to be the optimum mixture. Its resistivity was 19.37 Ω·m at 28 days under 25.31% porosity. The experimental results indicate that an ionically-conductive mortar can be produced by combining different air-entrainment agents with variable water-cement ratios to meet a specified electrical heating requirement.

## 1. Introduction

Conductive concrete is a functional constructive building material, and it can be used in deicing, snow-melting [1], electromagnetic shielding [2], and structural monitoring [3], etc. The conductive mechanism of traditional conductive concrete is conductive to circuits due to the interconnection of conductive materials with each other since the traditional conductive concrete normally is made by steel fiber [1], carbon fiber [4], graphite [5], and carbon nanotubes [6]. There are other kinds of new concrete that can have a relatively high conductivity, such as RPC (reactive powder concrete). The test results showed that the resistivity of RPC dropped from 1130 kΩ·cm to 137 kΩ·cm due to the presence of metallic fibers [7]. Therefore, the conductivity of these conductive concretes depends on the electrical properties of conductive materials and their distribution in the mixtures. There are many drawbacks in these conductive concretes, such as rusting of steel fiber [8] and twining of carbon fiber [9]. Ionically conductive mortar is carried out to overcome these drawbacks [10]. Previous experiments showed that ionically conductive mortar has good electric-heating performance [10,11]. It was intended to be used as a block partition wall for indoor-heating [12], so the most important performance of ionically conductive mortar is the electrical conductivity and heating efficiency. The porosity of ionically conductive mortar is about 20–35% to ensure its electric conductivity. Even though the ionically conductive mortar is a kind of multi-aperture material, the compressive strength of it is about 10–16 MPa [13], which is larger than the compressive strength of normal concrete hollow block stipulated by the national standard GB/T 13545-2014 [14]. High porosity means that more electrolyte solution can easily penetrate into mortar and consequently increase the conductivity and electrical-heating performance of mortar. On the other hand, high porosity also means the air inside the mortar will increase and the heat conductivity coefficient of either the solution or air is much lower than normal cement; thus the heat conductivity coefficient of ionically conductive mortar is low than normal cement which also means that the room made by ionically conductive mortar can have a better thermal isolation property once the room is heated.

The conductive mechanism of ionically conductive mortar is the directional migration of ions under external voltage which has been proved by many researchers [15,16,17]. The free ions and moisture inside the mortar is supplied by the electrolyte solution and water [10], and the mortar specimen is soaked in the electrolyte solution though connected pores during the fabricating process [11]. Thus, the number and shape of pores inside the mortar dictates the dispersion of the electrolyte within. A previous test also showed that the electric conductivity of ionically conductive mortar would increase sharply in 28 days, but the increasing rate became gentle with time [10,11]. To ensure the conductivity of ionically conductive concrete, several methods were raised such as using epoxy resin cover [10], embedding electrolyte supplement equipment [10], etc. The test results showed that all these methods could reduce the increasing rate of resistivity and finally get a relatively stable resistivity [10,11].

Cement-based composite is a kind of cellular material, and the porosity is generally about 10–15%. The pores inside the composite are categorized in connected pores and closed pores. The key factors influencing the porosity of cement-based composite include water–cement ratio and air entrainment [18,19]. Many researches have proved that the higher the water–cement ratio is, the higher the porosity in the cement-based composites [20,21,22]. However, the compressive strength of composites would decrease with increasing water–cement ratio. On the other hand, air-entraining agents have been used to disperse air bubbles in cement-based composites. Air-entraining agents could be categorized as chemical agents and physical admixtures [23]. Chemical agents react with cement to create bubbles [13]. For example, aluminum powder is the most common air-entraining chemical, generating hydrogen by reacting with calcium hydroxide during the hydration of cement [24]. The pores created by air-entraining chemical agent normally are communicating pores. Physical air-entraining admixtures generate air bubbles by decreasing the interfacial energy and surface tension of cement paste [25]. The pores created by physical air-entraining admixture normally are small and closed. It is plausible that large amounts of communicating pores could be generated by adding both chemical and physical agents.

Four kinds of air-entraining agents, aluminum powder, hydrogen peroxide, triterpenoid saponin (SJ-2), and lauryl sodium sulfate (K12), were singly added into the mortar specimens to investigate the porosity and pore shape. Further, aluminum powder was also mixed with an air-entraining admixture to increase the communicating porosity. Water–cement ratios were also varied to study the influence on pore structures and the resulting electric resistivity of the mortar.

## 2. Experiments

### 2.1. Materials for Test Specimens

All the materials used to fabricate the test specimens are shown in Table 1, Table 2 and Table 3.

### 2.2. Specimen Preparation

The mortar specimens were prepared according to the method of testing cements’ determination of strength (GB/T17671 1999) [26], with a 1:3 mass ratio of cement (grade PO325) to sand. The mass ratio of air-entraining agent and cement was determined based on the recommendation of the manufacturer which is 0.015% of SJ-2, 0.015% of K12, 0.075% of Aluminum powder, and 0.025 of Hydrogen peroxide (H_2_O_2_), respectively. A water–cement ratio in the range of 0.5 to 0.9 was used in the experiments. The designations of the specimens are shown in Table 4. Groups of each designation included three specimens, and the experiment results shown herein are the average of the three specimens.

The dimensions of the specimens were 40 mm × 40 mm × 40 mm. The fabrication process is summarized as follows: (1) the quantities of cement and sand were mixed in a mixer for 1 min; (2) tap water was added into the mixture and mixed for 1.5 min.; (3) air-entraining agent was added and mixed for 1 min.; (4) the mixture was cast into a mold and vibrated on a vibrating table for about 20 s; and (5) the surface of the specimen was finished with a steel trowel.

**Figure 1:**
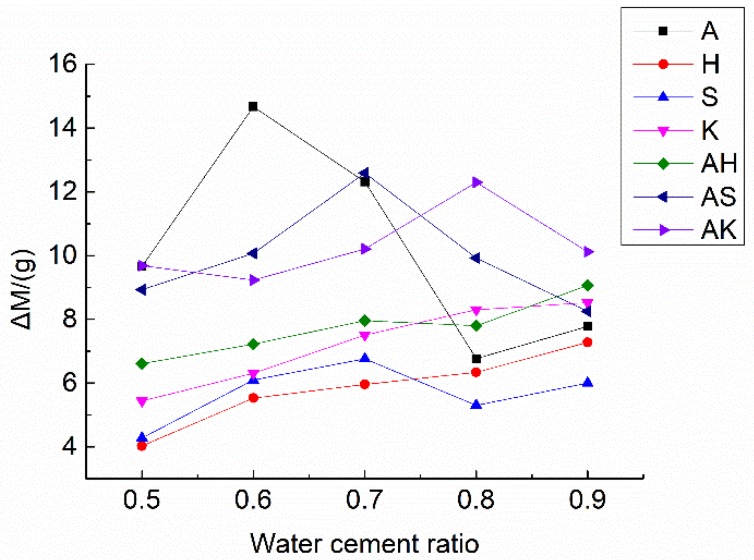
Changes in weight of specimens after being immersed.

The specimens were taken out of the mold after about 24 h and put into a curing box of 20 °C (68 °F) and 98% humidity for 28 days [27]. The specimens were dried in a vacuum-drying oven at 60 °C (140 °F) for 8 h and weighed after drying. Groups of three specimens were immersed in electrolyte solutions 4.8% CuSO_4_. Table 5 and Figure 1 show the weight change of each specimen in this process, where $M_{0}$ stands for the weight of the specimen before being immersed and $M_{1}$ is the weight on the day after, etc. The saturation limit of the weight increase rate of the specimen ΔM(%) calculated by Equation (1) was set to 0.025%, where $M_{i}$ is the weight of the specimen after being immersed for *i* days. If the specimen weight increase was lower than this limit, the immersion was stopped. All the specimens were taken out of the electrolyte after being immersed for 96 h to ascertain the saturation of the electrolyte. The increasing weight in this case is the electrolyte solution absorbed as well as the moisture content inside the mortar. According to the mechanism of ionically conductive mortar, a larger moisture content would provide better conductivity. The experimental results also proved this theory.

(1) $$\Delta M(\%)=\frac{M_{i+1}-M_{i}}{M_{i}}\times 100\%,\;i=0,\;1,\;2,\;3,\;4$$

**Figure 2:**
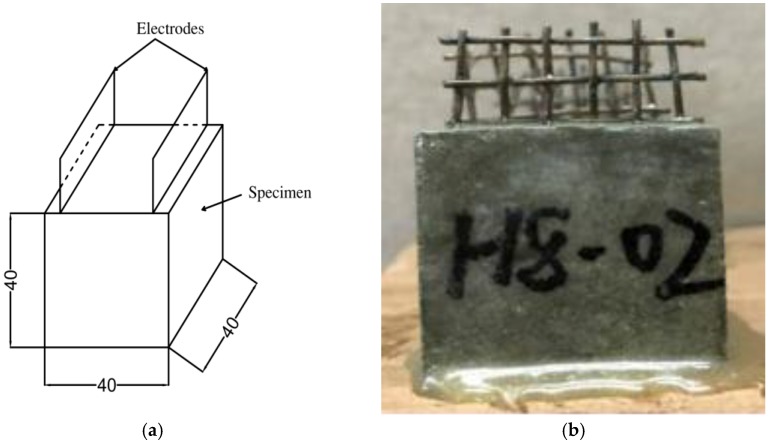
(**a**) The dimensions (in mm) of specimen; (**b**) photo of specimen.

After immersion was complete, the mortar specimens were wiped dry and coated with a 1 mm-thick layer of epoxy resin to prevent evaporation of moisture. Figure 2 shows a typical test specimen.

### 2.3. Measurement of Connected Porosity

Voids inside the mortar can be categorized into connected voids and closed voids. There was research that investigated the porosity and permeability of foam concrete [28]. Both water and steam were used to measure the permeability of foam concrete. The results showed that the weight of water soaked into concrete did not increase with increasing porosity, however, there was a significant increase in the steam permeating through the concrete [28]. This means that not all the voids inside the mortar can soak water, but only the connected voids will contribute to the weight of soaked water.

Many researchers used the air permeability to assess the permeability of concrete [29,30]. In this paper, the methanol method was adopted to evaluate the porosity of connected voids in the mortar specimen [31]. The processes of the methanol method are described as follows: (1) Submerge cement mortar specimens into the methanol solution with a molecular sieve for 5 days to dehydrate. The weight of each specimen overhanging in absolute methanol is denoted as *W*1. (2) Take out the specimens, and wipe and dry the surface. The specimen weight *W*2 is measured. (3) Put all the specimens in a vacuum drying oven to vacuumize and get rid of the methyl alcohol. Weigh a specimen every 1 to 2 h until the specimen weight does not change any more. Then the weight recorded is denoted as *W*3. The porosity, e, was calculated by Equation (2),

(2) $$e=\frac{(W2-W3)/p}{(W2-W1)/p}$$

where p is the specific gravity of methanol. According to the Equation (2), the p is not relevant to porosity e due to the elimination of p.

### 2.4. Measurement of Resistivity

**Figure 3:**
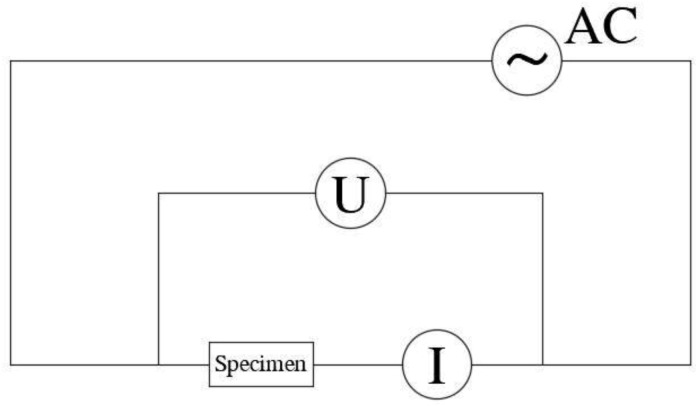
Testing circuit.

The resistivity of specimens was measured using a multi-meter according to the circuit shown in Figure 3. The experiments were carried out at 25 °C room temperature and 70% humidity. A 10 V AC power source was used for the experiments. The electrical resistivity *R* of a specimen was calculated by Equation (3).

(3) $$R=\frac{U}{I}\cdot \frac{A}{L}$$

where *U* is the voltage between the two ends of the specimen, *I* is the current of the circuit, and *A* and *L* are the cross-sectional area and length of the specimen, respectively.

## 3. Result and Discussion

### 3.1. Physical Characteristics

The 28 days compressive strength, conductivity and porosity of each group are shown in Table 6, where $\rho_{0}$ stands for the resistivity of the specimen on the day fabricated (the day specimens was finished by soaking the electrolyte solution and covering with epoxy resin); and $\rho_{1}$ is the resistivity on the day after, etc. The gradient of resistivity Δρ at 28 days can be calculated by Equation (4). The test results are presented as the average of three specimens. The coefficient of variation $c_{v}$ used to quantify the scatter of experimental data was set at 15%. If the $c_{v}$ of the compressive strength, the porosity or $\rho_{0}$ of any group of specimens was greater than 15%, that group of specimens would be re-fabricated.

(4) $$\Delta \rho =\frac{\rho_{28}-\rho_{0}}{\rho_{0}}\times 100\%$$

### 3.2. Influence of Air Entrainment and Water-Cement Ratio on the Porosity and Strength 

**Figure 4:**
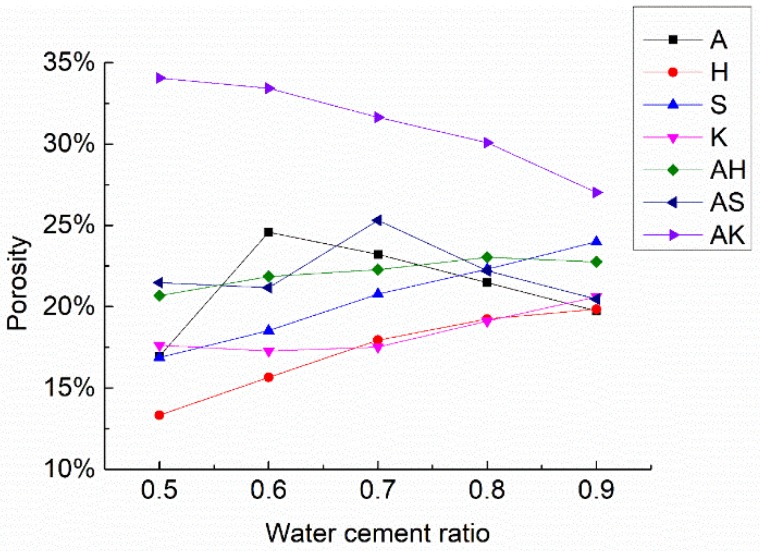
Changes in porosity of test specimens.

**Figure 5:**
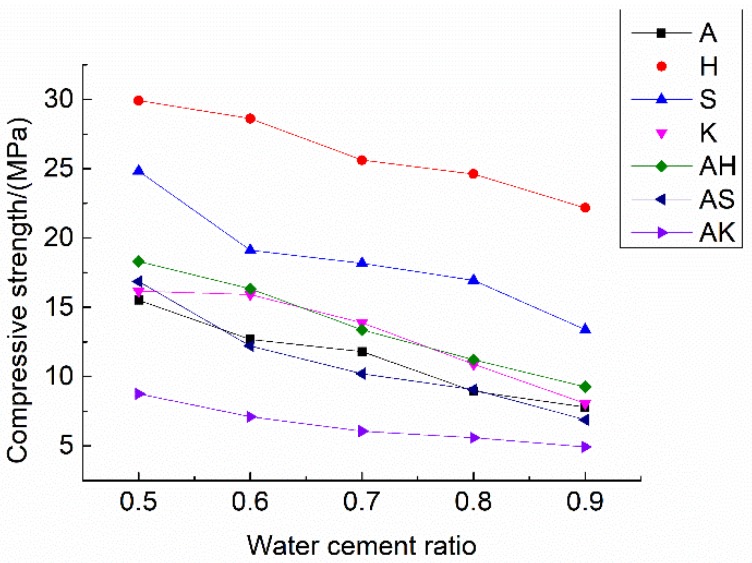
Changes in compressive strength with water–cement ratio.

The voids within the mortar can be categorized into connected voids and closed voids. Generally, the higher the water–cement ratio is, the higher the porosity is due to free water evaporation [20,21,22]. On the other hand, air-entraining agents can also be used to increase porosity. Both physical and chemical air-entraining agents were added in the mortar mixture to evaluate the porosity and strength of the specimens. As shown in Figure 4 and Figure 5, compressive strength decreases with the increasing water–cement ratio, while the porosity does not show a similar trend.

**Figure 6:**
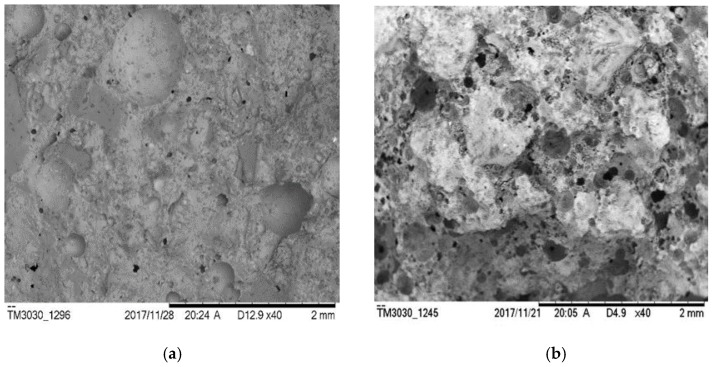
SEM photos of specimens with a single physical air-entraining agent (w/c = 0.5). (**a**) S-5; (**b**) K-5.

For the specimens with single physical air-entraining agent, the porosity of group S increased from 16.87% (w/c = 0.5) to 23.99% (w/c = 0.9), while compressive strength decreased from 24.84 MPa to 13.89 MPa. The porosity of group K increases from 17.63% (w/c = 0.5) to 20.60% (w/c = 0.9), while the compressive strength decreased from 16.17 MPa to 8.07 MPa. The porosity of the specimens with a single physical air-entraining agent rose monotonically with the increase of the water–cement ratio, but the ascending rate of group S is much higher than that of group K. The compressive strength of S group is much higher than the K group, even with the similar porosity. The admixture SJ-2 is a saponins air-entraining agent with large molecular weight [32]. The bubbles formed have a relatively thick membrane and good foam stabilization [33]. Figure 6a shows an SEM (scanning electron microscope) picture of a group S specimen. It can be seen that the pores are relatively small and uniform with relatively high compactness. The bubbles generated will not burst and remained with an increasing water–cement ratio. Hence, the porosity of group S specimens rapidly increased with the increasing water–cement ratio. This is also the reason why group S can achieve relatively high strength. The K12 admixture belongs to alkyl benzene sulfate foaming agent, which generates large amount of rich foam at a high foaming speed. However, its bubbles formation is not stable [34]. Small bubbles are easy to merge into relatively large ones and overflow. With the increase of the water–cement ratio, the bubbles generated by the K12 air-entraining agent burst in large amounts during vibration and their porosity did not increase significantly. As shown in Figure 6b, it is seen that part of the small bubbles merge into bigger ones, thus showing both large and small pores. The uneven pore structure and thin cement paste between pores caused a significant decrease in mortar strength.

**Figure 7:**
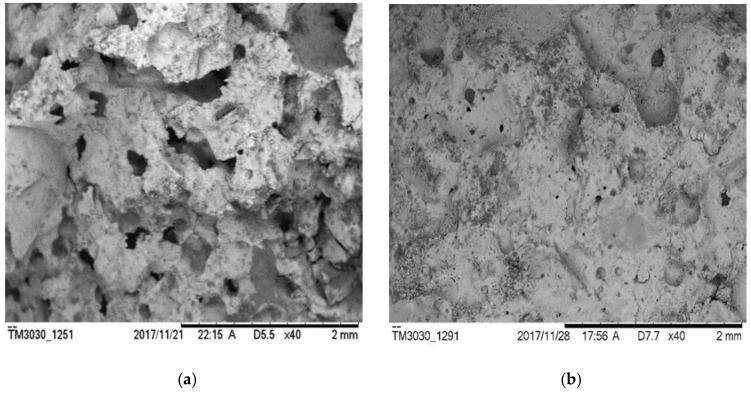
SEM photos of specimens with a single chemical air-entraining agent (w/c = 0.5). (**a**) A-5; (**b**) H-5.

**Figure 8:**
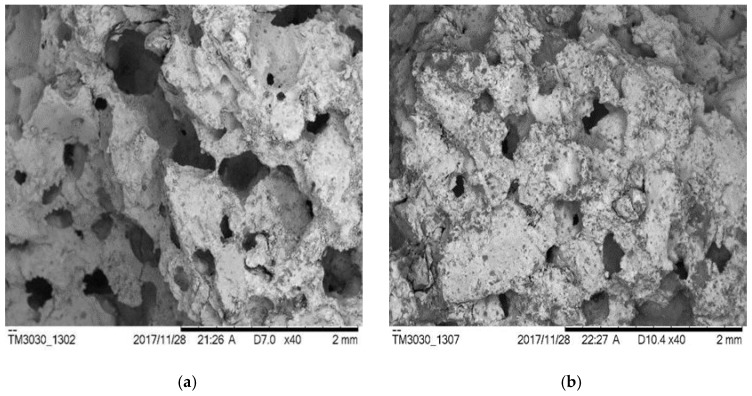
SEM photos of specimen with aluminum powder under different water–cement ratios. (**a**) A-7; (**b**) A-9.

The same observations also apply to group H with hydrogen peroxide. Group H has the highest strength of all specimens, because its overall porosity is relatively low due to the low amount of bubbles generated [35], as evidenced in Figure 7b. Aluminum powder has a relatively high efficiency of generating bubbles. It was observed during the experiment that the volume of the specimens expanded after the aluminum powder was mixed with cement. Large amounts of gas bubbles were evenly generated within the base materials. A vesicular structure with many interconnected pores was generated after hardening which effectively enhanced the porosity of the specimen, as evidenced in Figure 7. With the increased water–cement ratio, the porosity of group A rose from 16.96% (w/c = 0.5) to 24.58% (w/c = 0.6) and 23.21% (w/c = 0.7), respectively. When the water–cement ratio increased to 0.9, the porosity only rose to 19.74%. This is because when the water–cement ratio increased from 0.5 to 0.7, the fluidity of mortar would increase. Most of the slurry were more mobile than that with w/c = 0.5. Lots of gas would escape from the slurry, leading to high porosity as shown in Figure 8a. However, when the water–cement ratio was further increased, the slurry would be too thin. The setting speed of the slurry would lag behind the foaming speed of aluminum powder. Thus, it is difficult to stabilize bubbles which in turn decreases the porosity as shown in Figure 8b. But to those physical air entrainments (SJ-2 and K12), this rule does not exist. The porosity of specimens increased with the water–cement ratio as shown in Figure 4. This is because the foaming mechanisms between physical and chemical air entrainment are different. The physical air entrainment creates foam although it decreases the surface tension of moisture in mortar; however, the chemical air entrainment creates foam through the chemical reaction between the entrainment and mortar. So the character of the foam created by different air entrainments is totally different. The foam created by physical air entrainment was small and stable, it is hard to gather and form big bubbles to escape from the slurry due to the polar group absorbed to the surface of foam. However, the foam created by chemical air entrainment was big and unstable and can easily gather and escape from the slurry. So the mobility of slurry is much more sensitive to chemical air entrainment than physical air entrainment.

To aluminum powder, the results show that when the water–cement ratio is between 0.65 and 0.75, the optimal porosity can be achieved. However, the porosity decreased slightly when the water cement ratio was between 0.75 and 0.95. From a comprehensive analysis of the test results with air-entraining agents, group A with aluminum powder had the highest porosity. As a result, aluminum powder was used along with other air-entraining agents in the subsequent experiments to investigate the combined effect on air entrainment.

From the test results (i.e., Table 6 and Figure 4) of three groups of test specimens (group AH, AS and AK), the porosity of the specimen with mixed air entrainments is larger than those with single air entrainment. The porosity of these three groups did not increase when the water–cement ratio was increased from 0.5 to 0.9. However, the strength of the specimens simply decreased with the increase of the water–cement ratio. In group AH, the porosity stayed fairly unchanged with the highest point at w/c = 0.8. In group AS, the maximum porosity occurs at w/c = 0.7. In group AK, the porosity gradually declined with the increase of the water–cement ratio. This is because with the increase of the water–cement ratio, the mobility of base materials would increase. The expanding volume caused by the chemical reaction of aluminum powder reduced with the increasing water–cement ratio. When the water–cement ratio exceeded the limit value (0.8 for AH group, 0.7 for AS group, 0.5 for AK group), a small bubble caused by aluminum powder merged to form bigger bubbles and overflow, which led to the decrease of the overall porosity. However, with the increasing water–cement ratio, the moisture inside the mortar definitely increased. When the specimen was dried in the drying oven, the water inside the mortar become steam. This would cause the sharp increasing volume, and consequently, cause the micro-crack inside the mortar. This is the main reason why the strength of specimen sharply decreased with the increasing water–cement ratio.

**Figure 9:**
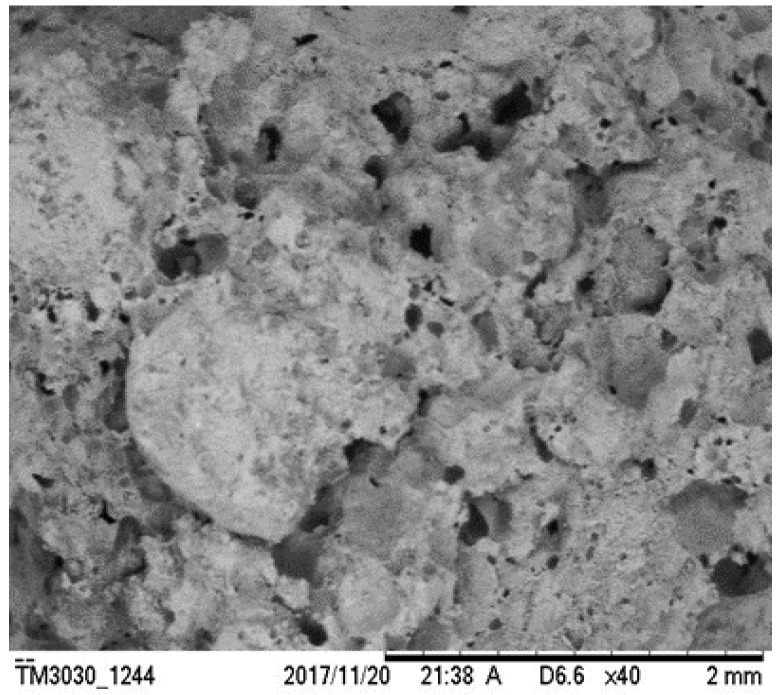
SEM photo of AK-7 specimen.

At the same time, test results showed that after mixing with another air-entraining agent, the optimal water–cement ratio for aluminum powder would vary by a certain degree. It is noted from Table 6 and Figure 4 that using another air-entraining agent in addition to aluminum powder greatly increases the porosity of the specimen, compared to the single air-entraining agent groups. Test results reveal that mixing aluminum powder can be introduced to interconnected pores based on single mixing to have interconnected pores and independent pores distribute uniformly. It improves the pore structure and pore diameter distribution, and further increases the porosity of the specimen. To verify this observation, scanning electron microscope was used to inspect group AK-5 specimens, as shown in Figure 9. It can be seen that there are not only parts of the interconnected pores, but also many evenly-located independent small pores on its surface. The independent bubbles were introduced by physical air-entraining agent K12, suggesting that the compound mixing of chemical and physical air-entraining agents can lead to a certain superimposed pores pattern. Therefore, the overall porosity can be increased based on single mixing, making the air-entraining effect superior to that using a single air-entraining agent. Among these three groups (AH, AS and AK), group AK has the highest porosity, but the compressive strength of AK group is lower than the allowable value of the Chinese national standard (GB/T 13545-2014) [14]; the specimens in group AS and AH both have porosity larger than 20%, but the AS7 has the highest porosity of 25.31% among the two groups; the compressive strength of AS7 is 10.21 MPa, which meets the Chinese national standard (GB/T 13545-2014) [14].

### 3.3. Resistivity Influenced by Air Entrainment and Water-Cement Ratio

**Figure 10:**
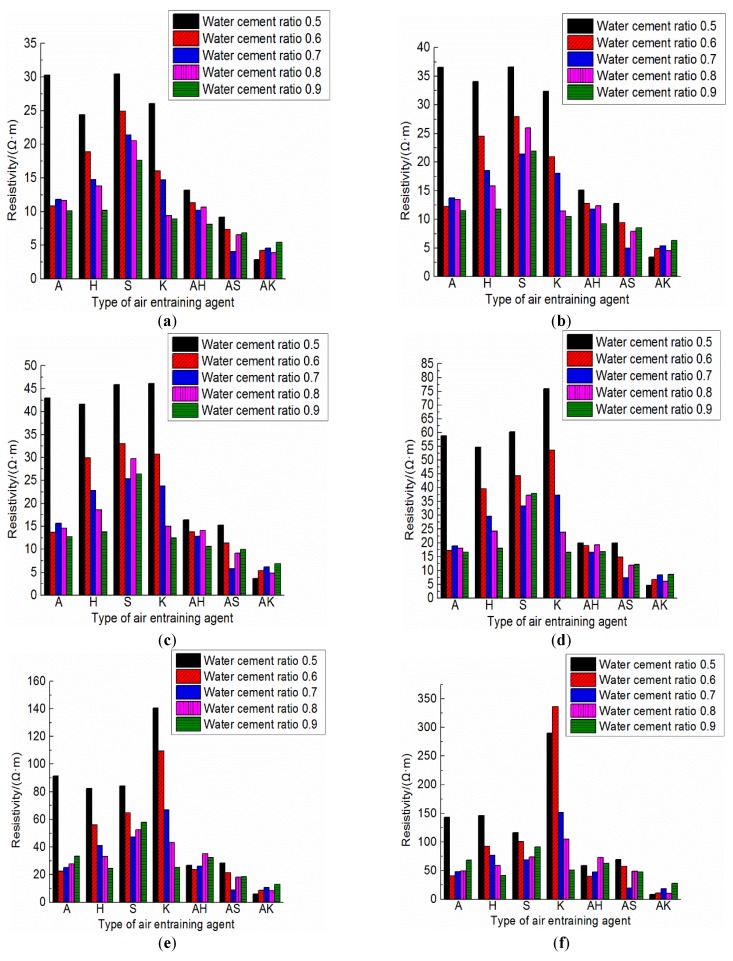
The resistivity of test specimens. (**a**) 0 day; (**b**) 1 day; (**c**) 3 days; (**d**) 7 days; (**e**) 14 days; (**f**) 28 days.

**Figure 11:**
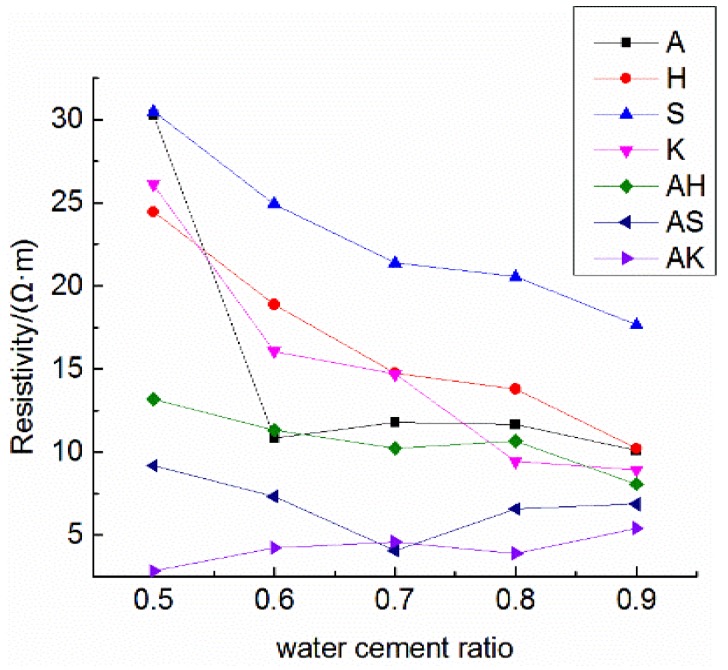
Changes in $\rho_{0}$ of specimens with water–cement ratio.

Dried and hardened cement paste and mortar have an electrical resistivity of approximately 104–107 Ω·m [36], which is decided by the constituents of cement, humidity, w/c ratio, etc., [37,38]. The resistivity of electrically conductive concrete is under 100 Ω·m [39], depending upon the electronic conduction within the conductive materials, such as steel fibers and graphite. In contrast, the conductivity of the ionically conductive mortar described in this paper solely depends upon the electrolyte dispersion within the mortar. Table 6 shows the resistivity of specimens at different ages, and the highest resistivity at 28 days of the test specimens is comparable to that of electrically conductive concrete. The resistivity of specimens with different air-entraining agents and water-cement ratios are compared in Figure 10. The changes in $\rho_{0}$ with water–cement ratios are shown in Figure 11. Figure 10 and Figure 11 clearly show the resistivity of specimens mixing with two air-entraining agents to be lower than that of specimens with a single air-entraining agent. And $\rho_{0}$ in the same group of specimens decreased with the increasing of porosity. With respect to the moisture absorption data shown in Table 5, it can be concluded that the specimen with higher porosity can absorb more electrolyte solution, and consequently, will have lower resistivity in most circumstances. For instance, from Table 5 and Table 6, the porosity of A5 and A6 are 16.96% and 24.58%; the electrolyte absorption of A5 and A6 are 9.66 g and 14.67 g; and the resistivity of A5 and A6 are 30.29 Ω·m and 10.82 Ω·m, respectively. When electrolyte solution could easily permeate into the mortar, the porosity of the mortar would increase and the resistivity of the mortar would decrease.

**Figure 12:**
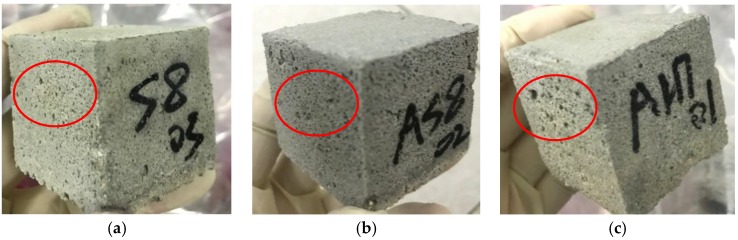
Apparent images of specimens. (**a**) S-8; (**b**) AS-8; (**c**) AH-7.

However, $\rho_{0}$ from different groups does not always decrease with the increasing of porosity. Three specimens, S-8, AS-8 and AH-7 are chosen herein for discussions. The porosity of these three specimens were 22.31%, 22.20% and 22.28%, respectively. However, the resistivity of these specimens were 20.54 Ω·m, 6.56 Ω·m and 10.21 Ω·m, respectively. The relation between pore structure and permeability of cement mortar was studied, and the results showed that the permeability did not just rely on porosity [40]. It also depends upon what causes the change of porosity, different water–cement ratios and/or hydration time [41]. In other words, it also relates to the pore aperture size, pore size distribution and pore pitch coefficient, etc. Figure 12 shows the apparent images of these three specimens. It can be seen in Figure 12 that the shape and distribution of the voids on the surface of the three specimens are totally different even if the porosity is approximately the same. Relative to another two specimens, S-8 is much more compact and less porous on the surface; it makes it more difficult for an electrolyte to penetrate and consequently yields the highest $\rho_{0}$ of the three specimens. AS-8 and AH-7 are specimens fabricated with two air-entraining agents; the porosity and resistivity of these two specimens are fairly close. However, as shown in Figure 12b,c, the aperture of the voids on the AH-7 surface appears to be bigger than that on the AS8 surface. Even though the number of voids on the AS-8 surface is seen to be more than that on the AH-7 surface, the cracks caused by connected voids are observed on the AS-8 surface. The electrolyte solution could penetrate into the mortar easily through these cracks, and this process caused the lower $\rho_{0}$ of AS-8 than AH-7.

$\rho_{28}$ is the resistivity of specimens at 28 days and Δρ is the gradient of resistivity at 28 days, which is defined in Equation (4). These two values indicate the availability of the electrolyte solution inside the specimens, and they are also an indicator of the stability of conductivity. There is a trend shown in Table 6 and Figure 10 that Δρ increases with decreasing of porosity. For example, most porosity of K group specimens are less than 20%, while the Δρ of K group specimens exceeds 1000%. Δρ of K-6 reached 1955.61%. AK group had the highest porosity of all specimens, the highest porosity of the AK group exceeded 30%, and the Δρ of AK group was also the lowest with Δρ only at 156.8%. It is noteworthy that this trend is not very obvious when the porosity of the specimens are approximately the same. This is because $\rho_{28}$ of ionically conductive mortar is influenced by not only porosity but also many other factors such as the degree of hydration of mortar, C/S and H/S ratios of hydration products, evaporation of water, and distribution of electrolyte within the mortar, etc., [40].

**Figure 13:**
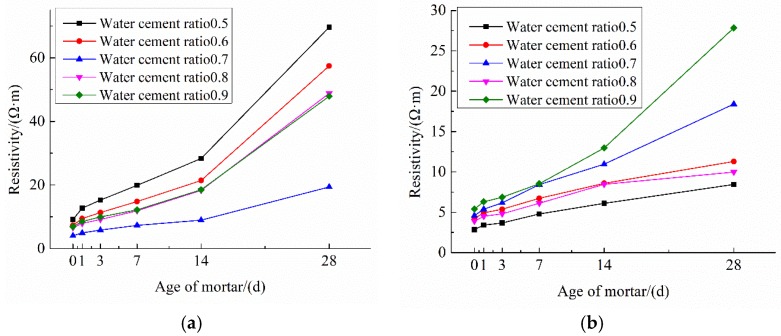
Changes in resistivity of specimens with mortar age. (**a**) AS group; (**b**) AK group.

**Figure 14:**
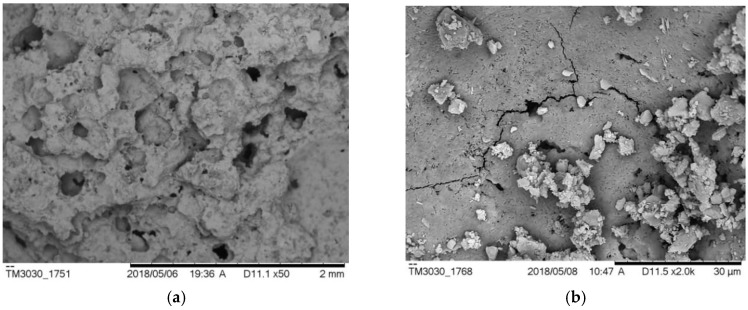

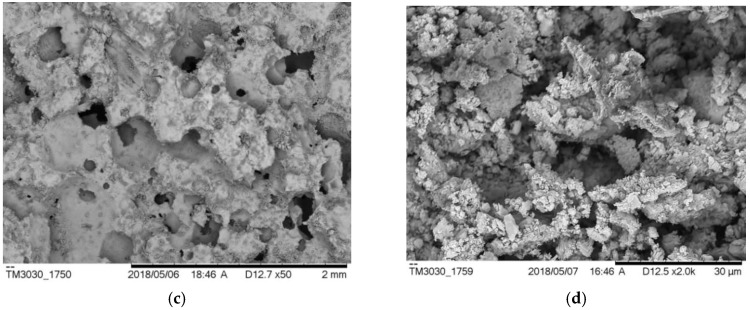
Microstructures of AK-5 and AS-7 Specimens. (**a**) Magnified 50 time of AK-5; (**b**) Magnified 2000 time of AK-5; (**c**) Magnified 50 time of AS-7; (**d**) Magnified 2000 time of AS-7.

To determine the optimized water–cement ratio and combination of air-entraining agents, the AS and AK groups were chosen to compare for their relatively low $\rho_{0}$ and $\rho_{28}$. Figure 13 shows the changes of these two groups’ resistivity with ages. Both AS-7 and AK-5 had excellent $\rho_{0}$ and $\rho_{28}$, and those values also met the demand of traditional conductive concrete [39]. AK-5 had the lower resistivity, with $\rho_{28}$ = 8.25 Ω·m. The microstructure images of these specimens are compared in Figure 14. Figure 14a,c shows the images of the two specimens magnified 50 times, and the images show that pore structure and numbers are similar. However, when showing these images magnified 2000 times, different results emerge. Figure 14b shows that there are many micro-cracks inside AK-5 and hydration products C-S-H (calcium silicate hydrate) which contribute to most of the strength of mortar, much less than those in AS-7 as shown in Figure 14d. In addition, there are some Ca(OH)_2_, which are hexagonal crystals dispersed inside the mortar. This component is easily broken when the specimen is stressed under loading. As a result, it can be predicted that the strength of AK-5 specimens would be low due to these drawbacks. The number and structure of C-S-H inside the AS-7 shows that the hydration process is fully developed and there are no micro-cracks observed. Therefore, the AS-7 specimen which is fabricated with aluminum powder and SJ-2 air-entraining agent (with w/c = 0.7) was determined to be the optimized mixing ratio for heating applications.

## 4. Conclusions

In this study, 35 groups of specimens were fabricated with different air-entraining agents as well as with variable water–cement ratios. The porosity and resistivity of specimens were compared to investigate the influence of air entrainment and water–cement ratio on the performance of ionically conductive mortar, and the following conclusions are drawn:

1. Mixing with a single air-entraining agent can increase the porosity and permeability of ionically conductive mortar and eventually improve the conductivity of mortar. According to the results of SEM results, different air-entraining agents create different types of pores. The physical air-entraining agent mostly creates closed voids and the chemical air-entraining agent created connected voids. The porosity of most specimens increased with the increasing water–cement ratio. For the specimens fabricated with aluminum powder, the optimized water–cement ratio is about 0.6–0.7. The specimens fabricated with aluminum powder and this water–cement ratio have the highest porosity of connected voids compared with other specimens fabricated with a single air-entraining agent.
2. Based on the preliminary experimental results, aluminum power was chosen to mix with other air-entraining agents to add to the mortar mixture to increase porosity. The results showed that the porosity of specimens with two different air-entraining agents is much higher than the specimens with a single air-entraining agent. SEM experiments confirmed that both connected and closed voids existed inside the mortar. The water–cement ratio was optimized when aluminum powder was used as an air-entraining agent. This optimized ratio changed when a different air-entraining agent was also added to the mixture. The optimized w/c is about 0.8 for the AH group, 0.7 for the AS group, and 0.5 for the AK group, respectively.
3. The conductivity of specimens with two different air-entraining agents is significantly better than the specimens with a single air-entraining agent. The $\rho_{0}$ of specimens in the same group decreased with increasing porosity. This is because the conductivity of ionically conductive mortar takes the most advantage of the permeability of specimens. The higher the porosity is, the easier it is for the electrolyte to penetrate, with other conditions being equal. However, $\rho_{0}$ of the specimens from different groups did not always decrease with increasing porosity. Key factors which influenced the permeability of the mortar also included the characteristic of the voids inside the mortar such as category, dimension, distance between voids, etc.
4. The conductivity of the ionically conductive mortar at 28 days meets the requirement of normal electrically conductive concrete. For the gradient of resistivity at 28 days, Δρ increases generally with the decreasing of porosity. This trend is not always obvious. This is because that $\rho_{28}$ is influenced not only by porosity, but also by many other factors such as the hydration degree of the mortar, C/S and H/S ratios of the hydration products, evaporation of water, and distribution of electrolyte solution inside the mortar, etc.
5. The $\rho_{0}$, $\rho_{28}$ and micro-image of AK-5 and AS-7 were analyzed. Even though the AK5 has lower $\rho_{0}$ and $\rho_{28}$, AS-7 was chosen to be the optimized mixing ratio. This is because that micro-image shows that the hydration process of AS-7 is complete. There are several drawbacks of AK-5, such as micro-cracks and more Ca(OH)_2_ crystals which would decrease the mechanical strengths of the mortar significantly.

## Figures and Tables

**Table 1:** Material properties.

Materials	Properties of Materials
Cement	Portland cement PO325, ignition loss 2.28%, initial setting time ≥ 45 min, chemical composites are shown in Table 2
Sand	Ordinary river sand (SiO_2_), 50% of the total mass particle size ≤ 0.25 mm, the average particle size: 0.25–0.5 mm
Water	Ordinary tap water, composites are shown in Table 3
Copper electrode	Diameter 1 mm, aperture 5 mm × 5 mm, processed into a size of 40 mm × 65 mm sheet as the electrode
CuSO_4_	Electrolyte solution for immersing the specimens; Analytical reagent; content ≥ 99%
Aluminum powder	99.5% purity, diameter 60–75 µm
Triterpene saponin air-entraining admixture SJ-2	Light yellow powder; content of natural triterpene saponin ≥ 63%
Hydrogen peroxide	Liquid, 30% purity
Sodium lauryl sulfate air-entraining admixture K12	White powder, content of active substance > 94%
Anhydrous methanol	99.9% purity
Type 3A molecular sieve	SiO_2_/Al_2_O_3_ ≈ 2, effective aperture is about 0.3 nm, diameter 1.7–2.5 mm, water absorption > 80%(weight)
Epoxy resin	AB Type epoxy resin. Part A is resin. Part B is hardener. Part A and Part B mixed by mass ratio 1:1. Initial set time 3 min, final set time 5–10 min.

Note: 1 MPa = 145 psi; 1 in. = 25.4 mm.

**Table 2:** Cement chemical composite.

Composites	CaO	SiO_2_	Al_2_O_3_	Fe_2_O_3_	MgO	K_2_O	Na_2_O	SO_3_	Cl^−^
Content (%)	62.17	21.84	6.56	4.15	2.23	0.34	0.41	2.26	0.013

**Table 3:** Water composite (pH = 7.56).

Composites	Al	Fe	Mn	Cu	Zn	Cl^−^	SO_4_^−^	NO_3_^−^	As	Cr(VI)	Se
Content (mg/L)	0.03	<0.05	<0.05	<0.05	<0.05	8.4	29.9	0.62	<0.001	<0.004	<0.001

**Table 4:** Designations of Specimens.

Air-Entraining Agent	Water/Cement Ratio				
	0.5	0.6	0.7	0.8	0.9
Reference mortar	-	-	B	-	-
Aluminum powder	A-5	A-6	A-7	A-8	A-9
Hydrogen peroxide (H_2_O_2_)	H-5	H-6	H-7	H-8	H-9
SJ-2 air-entraining agent	S-5	S-6	S-7	S-8	S-9
K12 air-entraining agent	K-5	K-6	K-7	K-8	K-9
Aluminum + H_2_O_2_	AH-5	AH-6	AH-7	AH-8	AH-9
Aluminum + SJ-2	AS-5	AS-6	AS-7	AS-8	AS-9
Aluminum + K12	AK-5	AK-6	AK-7	AK-8	AK-9

**Table 5:** Changes in weight during immersion process.

Specimens	Weight (g)					d*M* (g)
	(0d) *M*0	(1d) *M*1	(2d) *M*2	(3d) *M*3	(4d) *M*4	
B	145.17	147.77	148.02	148.24	148.29	3.12
A5	138.98	147.92	148.50	148.54	148.64	9.66
A6	127.08	140.33	140.95	141.47	141.75	14.67
A7	131.13	142.23	142.94	143.29	143.44	12.31
A8	138.82	144.48	144.83	145.30	145.59	6.77
A9	128.34	134.95	135.26	135.79	136.12	7.78
H5	146.09	149.75	150.07	150.08	150.12	4.03
H6	149.72	155.01	155.21	155.23	155.25	5.53
H7	150.60	156.34	156.53	156.55	156.56	5.96
H8	148.01	154.12	154.33	154.34	154.35	6.34
H9	143.87	150.86	151.07	151.13	151.15	7.28
S5	145.82	149.50	149.84	149.99	150.10	4.28
S6	134.03	139.19	139.72	139.95	140.13	6.10
S7	138.38	144.21	144.74	145.00	145.14	6.76
S8	140.00	144.74	144.92	145.12	145.30	5.30
S9	131.25	136.66	136.88	137.14	137.25	6.00
K5	120.84	125.25	125.72	126.19	126.28	5.44
K6	116.72	122.34	122.67	123.01	123.03	6.31
K7	125.48	132.53	132.83	132.96	132.99	7.51
K8	137.73	145.75	145.87	145.99	146.03	8.30
K9	142.80	151.10	151.19	151.29	151.32	8.52
AH5	128.12	133.59	134.03	134.58	134.73	6.61
AH6	132.02	137.86	138.51	139.03	139.24	7.22
AH7	130.88	137.55	138.09	138.62	138.84	7.96
AH8	128.41	134.93	135.45	136.01	136.21	7.80
AH9	128.25	136.18	136.66	137.15	137.31	9.06
AS5	124.44	131.86	132.54	133.28	133.37	8.93
AS6	124.66	132.93	133.67	134.66	134.73	10.07
AS7	113.45	124.29	125.11	125.92	126.04	12.59
AS8	127.94	136.32	136.94	137.76	137.86	9.92
AS9	130.21	137.15	137.66	138.37	138.46	8.25
AK5	112.59	120.97	121.49	122.00	122.27	9.68
AK6	111.98	118.99	120.12	121.00	121.21	9.23
AK7	116.69	125.35	126.02	126.68	126.90	10.21
AK8	126.27	136.99	137.68	138.25	138.57	12.30
AK9	127.38	136.20	136.75	137.25	137.50	10.12

Note: $dM=M_{4}-M_{0}$.

**Table 6:** Changes in resistivity and porosity of specimens with time lapsed.

Specimens	28 days Compressive Strength (MPa)	Porosity	Resistivity (Ω·m)						(Δρ)
			0 day $\left(\rho_{0}\right)$	1 day $\left(\rho_{1}\right)$	3 day $\left(\rho_{3}\right)$	7 day $\left(\rho_{7}\right)$	14 day $\left(\rho_{14}\right)$	28 day $\left(\rho_{28}\right)$	
B	29.28	17.34%	11.839	23.711	40.491	56.549	93.039	159.819	1249.94%
A-5	15.50	16.96%	30.293	36.563	42.962	58.827	91.261	143.113	372.42%
A-6	12.68	24.58%	10.824	12.242	13.737	17.153	22.341	40.833	277.25%
A-7	11.80	23.21%	11.798	13.732	15.660	18.847	25.241	48.465	310.78%
A-8	8.94	21.48%	11.656	13.452	14.625	18.033	27.654	49.452	324.25%
A-9	7.79	19.74%	10.112	11.509	12.740	16.589	33.256	68.103	573.47%
H-5	29.92	13.33%	24.443	34.048	41.608	54.750	82.319	146.303	498.55%
H-6	28.62	15.65%	18.878	24.541	29.917	39.621	55.883	92.332	389.08%
H-7	25.62	17.94%	14.757	18.511	22.805	29.677	41.251	76.867	420.88%
H-8	24.61	19.24%	13.798	15.812	18.552	24.237	33.156	58.704	325.44%
H-9	22.17	19.84%	10.199	11.757	13.762	17.956	24.529	41.839	310.23%
S-5	24.84	16.87%	30.494	36.631	45.845	60.320	84.289	116.051	280.57%
S-6	19.12	18.52%	24.934	27.907	32.960	44.356	64.615	100.891	304.63%
S-7	18.18	20.77%	21.376	21.385	25.388	33.378	47.308	69.136	223.43%
S-8	16.94	22.31%	20.544	25.960	29.778	37.194	52.588	73.755	259.01%
S-9	13.39	23.99%	17.652	21.932	26.433	37.933	57.744	91.537	418.58%
K-5	16.17	17.63%	26.091	32.329	46.108	75.923	140.526	290.259	1012.49%
K-6	15.92	17.27%	16.051	20.964	30.746	53.615	109.450	336.376	1995.61%
K-7	13.87	17.52%	14.699	18.063	23.761	37.266	66.970	152.084	934.68%
K-8	10.90	19.12%	9.439	11.441	14.952	23.817	43.265	105.073	1013.22%
K-9	8.07	20.60%	8.926	10.479	12.435	16.557	25.060	50.711	468.09%
AH-5	18.31	20.68%	13.165	15.087	16.360	19.940	26.614	59.246	350.02%
AH-6	16.33	21.86%	11.305	12.726	13.747	18.952	23.925	40.292	256.40%
AH-7	13.38	22.28%	10.215	11.746	12.816	16.589	26.127	47.581	365.79%
AH-8	11.22	23.04%	10.660	12.370	14.077	19.252	35.343	73.361	588.19%
AH-9	9.26	22.75%	8.080	9.249	10.552	16.736	32.336	62.535	673.91%
AS-5	16.87	21.47%	9.172	12.724	15.262	19.896	28.336	69.629	659.17%
AS-6	12.21	21.17%	7.328	9.442	11.323	14.808	21.413	57.459	684.05%
AS-7	10.21	25.31%	4.042	4.955	5.821	7.283	8.945	19.375	379.29%
AS-8	9.06	22.20%	6.566	7.920	9.136	11.898	18.274	48.843	643.85%
AS-9	6.88	20.47%	6.866	8.539	9.944	12.174	18.489	47.910	597.82%
AK-5	8.75	34.05%	2.852	3.408	3.681	4.792	6.109	8.452	196.35%
AK-6	7.10	33.43%	4.235	4.931	5.376	6.719	8.588	11.288	166.53%
AK-7	6.06	31.64%	4.589	5.377	6.174	8.434	10.959	18.399	300.96%
AK-8	5.58	30.08%	3.888	4.507	4.810	6.123	8.455	9.984	156.80%
AK-9	4.93	27.02%	5.417	6.295	6.855	8.538	12.956	27.832	413.80%
